# Novel Labdane Diterpenes-Based Synthetic Derivatives: Identification of a Bifunctional Vasodilator That Inhibits Ca_V_1.2 and Stimulates K_Ca_1.1 Channels

**DOI:** 10.3390/md20080515

**Published:** 2022-08-13

**Authors:** Gabriele Carullo, Simona Saponara, Amer Ahmed, Beatrice Gorelli, Sarah Mazzotta, Alfonso Trezza, Beatrice Gianibbi, Giuseppe Campiani, Fabio Fusi, Francesca Aiello

**Affiliations:** 1 Department of Life Sciences, University of Siena, Via Aldo Moro 2, 53100 Siena, Italy; 2 Department of Chemistry, University of Milan, Via Golgi 19, 20133 Milano, Italy; 3 Department of Biotechnology, Chemistry and Pharmacy, University of Siena, Via Aldo Moro 2, 53100 Siena, Italy; 4 Department of Pharmacy, Health and Nutritional Sciences, University of Calabria, Edif. Polifunzionale, 87036 Arcavacata di Rende, Italy

**Keywords:** sclareolide, labdane, hypertension, vasorelaxant activity, Ca_V_1.2 channels, K_Ca_1.1 channels, Langendorff perfused heart, docking simulations, molecular dynamics simulations

## Abstract

Sesquiterpenes such as leucodin and the labdane-type diterpene manool are natural compounds endowed with remarkably in vitro vasorelaxant and in vivo hypotensive activities. Given their structural similarity with the sesquiterpene lactone (+)-sclareolide, this molecule was selected as a scaffold to develop novel vasoactive agents. Functional, electrophysiology, and molecular dynamics studies were performed. The opening of the five-member lactone ring in the (+)-sclareolide provided a series of labdane-based small molecules, promoting a significant in vitro vasorelaxant effect. Electrophysiology data identified **7** as a Ca_V_1.2 channel blocker and a K_Ca_1.1 channel stimulator. These activities were also confirmed in the intact vascular tissue. The significant antagonism caused by the Ca_V_1.2 channel agonist Bay K 8644 suggested that **7** might interact with the dihydropyridine binding site. Docking and molecular dynamic simulations provided the molecular basis of the Ca_V_1.2 channel blockade and K_Ca_1.1 channel stimulation produced by **7**. Finally, **7** reduced coronary perfusion pressure and heart rate, while prolonging conduction and refractoriness of the atrioventricular node, likely because of its Ca^2+^ antagonism. Taken together, these data indicate that the labdane scaffold represents a valuable starting point for the development of new vasorelaxant agents endowed with negative chronotropic properties and targeting key pathways involved in the pathophysiology of hypertension and ischemic cardiomyopathy.

## 1. Introduction

Cardiovascular disorders represent the principal cause of death worldwide. Hypertension is the main risk factor for myocardial infarction, endothelial dysfunction, and metabolic syndrome [1]. Successful antihypertensive therapies exist; however, they are characterized by important side effects, limiting patient compliance. In the last decade, the discovery of new drugs has experienced a significant decrease, despite the growing investments in pharmaceutical research and development. This favored the progressive engagement of natural products in preventing/treating hypertension and several other diseases [2,3,4,5,6,7,8,9,10]. A great number of plant-derived extracts and substances, such as polyphenols, display vasorelaxant activity in in vivo and ex vivo models by modulating K_Ca_1.1 and/or Ca_V_1.2 channels, as well as by promoting NO synthesis [11,12,13,14]. The research on novel, naturally occurring, vasoactive agents, which is rapidly expanding, also involves sesquiterpene lactones and labdane diterpenoids [15]. In particular, sesquiterpene lactones can induce both endothelium-independent and -dependent relaxation, the latter being mediated by NO production [16].

**Figure 1:**
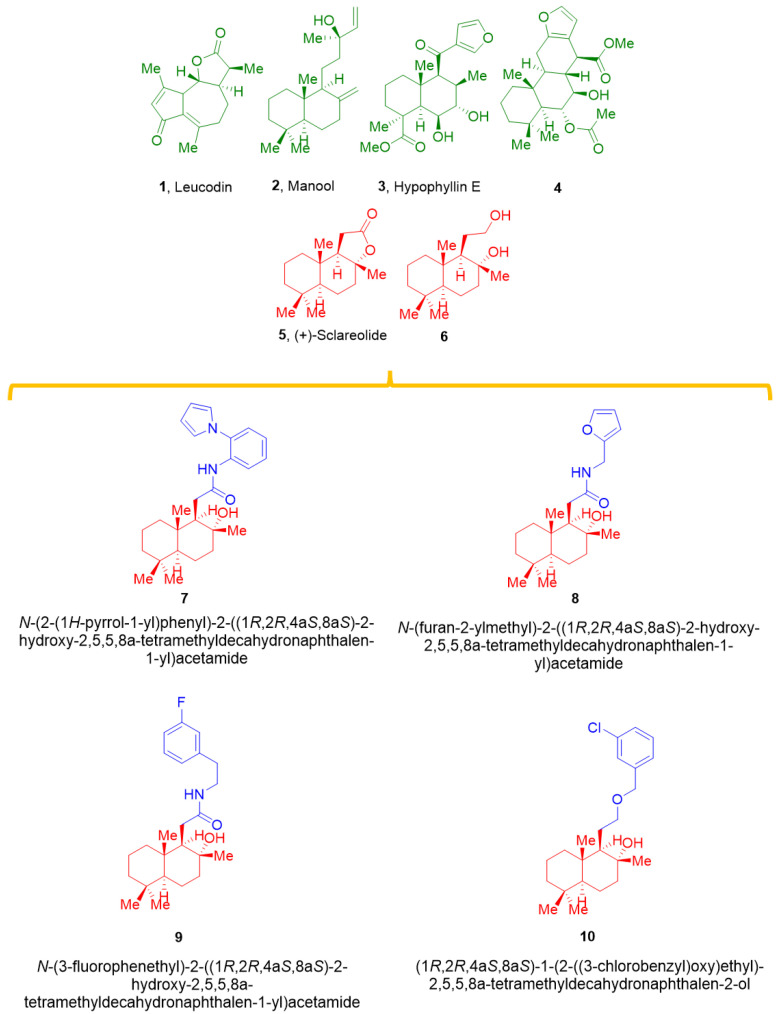
Labdane diterpenes vasorelaxant agents (**1**–**4**), (+)-sclareolide (**5**), sclareol (**6**), and new semi-synthetic tools (**7**–**10**).

Furthermore, activation of the endothelial NO-cGMP pathway, opening of K^+^ channels, modification of intracellular Ca^2+^ mobilization [17], and adenylyl cyclase activation [18] underpin labdanes’ cardiovascular actions. Specifically, sesquiterpene leucodin (1, Figure 1) relaxed rat aorta rings, blocking Ca_V_1.2 channels and promoting endothelium-dependent NO release [19]. The latter has been associated with the antihypertensive effect exerted by the labdane-type diterpene manool (2, Figure 1), which only promotes the relaxation of endothelium-intact rings [20]. 

Interestingly, hypophyllins extracted from the aerial parts of *Hypoestes phyllostachya* “Rosea” showed potent vasorelaxant activity on endothelium-intact thoracic aorta rings precontracted by KCl, which allowed the identification of the X-ray structure of hypophyllin E (3, Figure 1) [21]. Finally, the diterpene methyl-6α-acetoxy-7β-hydroxyvouacapan-17 β-oate (4, Figure 1) decreased the ionic current through Ca_V_1.2 channels in freshly dissociated vascular smooth muscle cells [22]. From a medicinal chemistry point of view, synthetic derivatives of natural flavonoids can interfere with K_Ca_1.1 and/or Ca_V_1.2 channels with different degrees of activity and multifunctional profiles [23,24]. Recently, the synthesis of new labdane-based diterpenes (6–10, Figure 1), acting as transient receptor potential channel subfamily V member 4 (TRPV4) antagonists, was described in [25]. Given the structural similarity among the vasoactive compounds 1–4 and the naturally occurring sesquiterpene lactone (+)-sclareolide (5) and its derivatives 6–10 (Figure 1), an in-depth in vitro and in silico investigation of their potential vasorelaxant activity was performed. The results demonstrate that derivatization of the **5** generated molecules was capable of inhibiting Ca_V_1.2 and stimulating K_Ca_1.1 channels, with **7** being the most promising bi-functional vasodilator.

## 2. Results

### 2.1. Effect of ***5*** and Its Derivatives on High KCl-Induced Contraction and Ba^2+^ Current through Ca_V_1.2 Channels (I_Ba1.2_)

**Figure 2:**
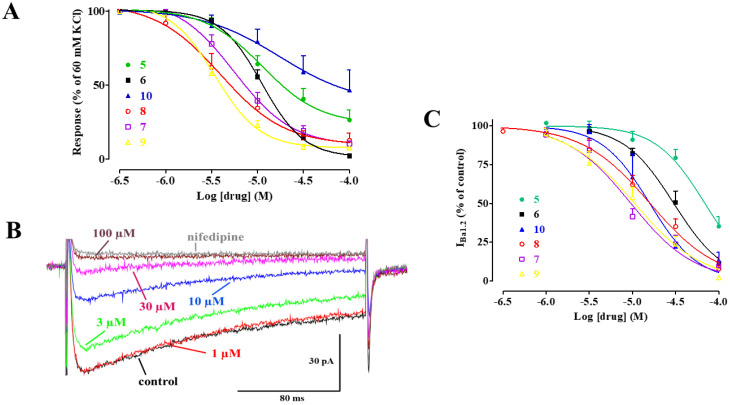
Effect of **5** and its derivatives on high KCl-induced contraction and I_Ba1.2_. (**A**) Concentration-response curves constructed in endothelium-denuded rings pre-contracted by 60 mM KCl. In the ordinate scale, the response is reported as a percentage of the initial tension induced by KCl. Data points represent the mean ± s.e.m. (*n* = 3–7). (**B**) Traces of I_Ba1.2_, recorded from a single rat tail artery myocyte, elicited by 250-ms clamp pulses to 10 mV from a V_h_ of −50 mV, measured in the absence (control) or presence of cumulative concentrations of **7**. The block caused by 10 µM nifedipine is also shown. (**C**) Concentration–response curves constructed in tail artery myocytes. On the ordinate scale, the current amplitude is reported as a percentage of the value recorded just before the addition of the first concentration of each compound. Data points represent the mean ± s.e.m. (*n* = 5–8).

**Figure 3:**
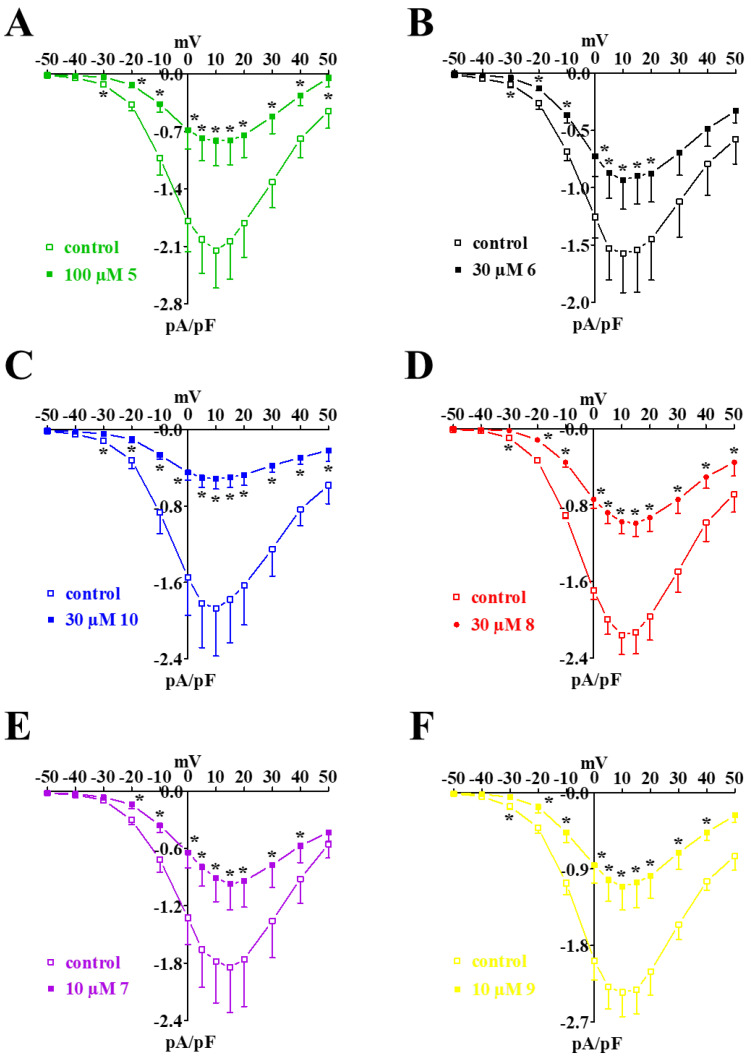
Current–voltage relationships of I_Ba1.2_ inhibition induced by **5** and its derivatives in single rat tail artery myocytes. (**A**–**F**) Current-voltage relationships, recorded from a V_h_ of −50 mV, constructed prior to the addition (control) and in the presence of (**A**) 100 µM **5**, (**B**) 30 µM **6**, (**C**) 30 µM **10**, (**D**) 30 µM **8**, (**E**) 10 µM **7**, and (**A**) 10 µM **9**. Data points are mean ± s.e.m. (*n* = 5–6). * *p* < 0.05 vs. control, Student’s t test for paired samples.

A series of experiments was performed to assess the effect of **5** and its derivatives, namely **6**, **7**, **8**, **9**, and **10**, on electromechanical coupling. Rings were depolarized using 60 mM KCl; when muscle tone reached a plateau, each compound was added cumulatively. As shown in Figure 2A, all the compounds caused a concentration-dependent relaxation of depolarized aorta preparations, with IC_50_ values ranging between 5 µM and 69 µM, and E_max_ values ranging between 53% and 99% (Table 1). In a second series of experiments, the effect of each compound on I_Ba1.2_ was evaluated. Figure 2B shows illustrations of the inward current elicited by a clamp pulse to 10 mV, from a V_h_ of −50 mV under control conditions and after the addition of cumulative concentrations of **7**, which inhibited peak I_Ba1.2_ in a concentration-dependent manner. Similar results were obtained with the other molecules (Figure 2C), with IC_50_ values ranging between 13 µM and 69 µM, and E_max_ values ranging between 65% and 98% (Table 1). The current–voltage relationships, shown in Figure 3, demonstrated that **5** and its derivatives significantly decreased the peak I_Ba1.2_ in a wide range of membrane potential values, without affecting either the apparent maximum at 10–15 mV or the threshold at approximately −40 mV.

### 2.2. Effect of ***5***, ***7***, ***8***, and ***9*** on the Contraction Induced by Moderate KCl Concentrations

**Figure 4:**
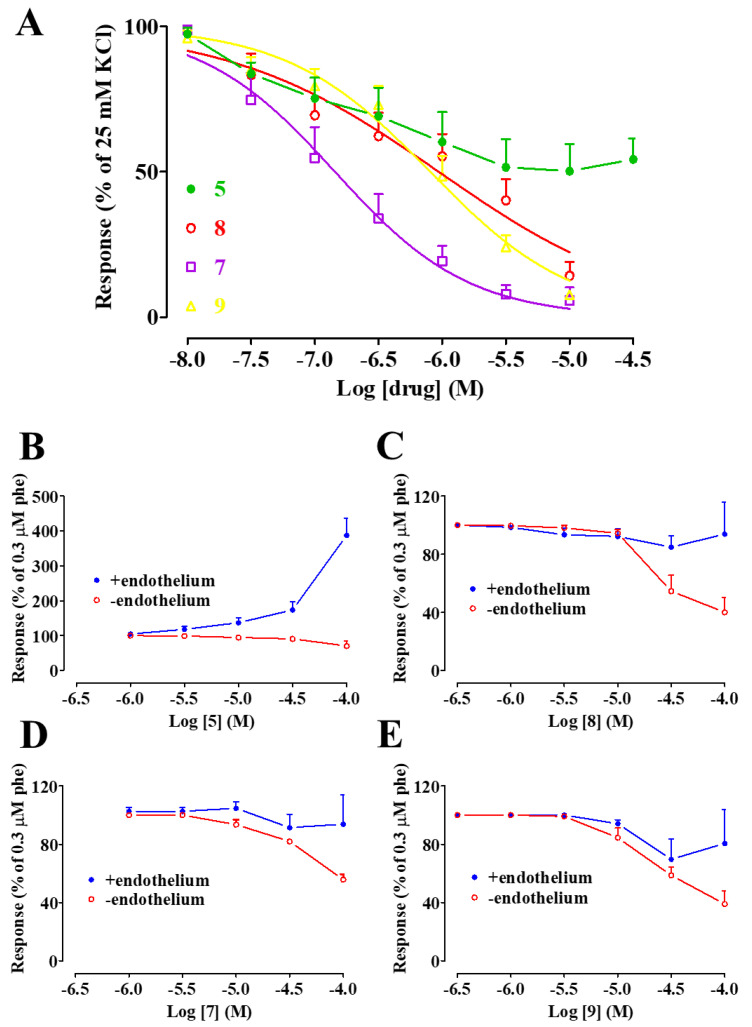
Effect of **5** and its derivatives on moderate concentration of KCl- or phenylephrine-induced contractions. (**A**) Concentration–response curves constructed in endothelium-denuded rings pre-contracted by 25 mM KCl. In the ordinate scale, the response is reported as a percentage of the initial tension induced by KCl. Data points represent the mean ± s.e.m. (*n* = 3–8). (**B**–**E**) Concentration–response curves for (**B**) **5**, (**C**) **8**, (**D**) **7**, and (**E**) **9** constructed in rings either endothelium-denuded or -intact, pre-contracted by 0.3 µM phenylephrine. In the ordinate scale, the response is reported as a percentage of the initial tension induced by phenylephrine (phe). Data points represent the mean ± s.e.m. (*n* = 5–7).

In this series of experiments, **5** and the three most effective derivatives according to the previous assays, were assessed on rings depolarized by a moderate increase in the extracellular KCl concentration. It was found that **7**, **8**, and **9**, as well as **5,** though to a lesser extent, relaxed the contraction evoked by 25 mM KCl (Figure 4A) with IC_50_ values ranging between 0.2 µM and 2 µM, and E_max_ values ranging between 46% and 94% (Table 1).

### 2.3. Effect of ***5***, ***7***, ***8***, and ***9*** on Phenylephrine-Induced Contraction

This series of experiments was performed to investigate the effect of the compounds on the pharmaco-mechanical coupling. In endothelium-denuded rings, **5**, **7**, **8**, and **9** reverted the α_1_ adrenergic receptor agonist phenylephrine-induced contraction in a concentration-dependent manner (Figure 4B–E), though their efficacy never exceeded 60% (Table 1). The presence of an intact endothelium counteracted the vasorelaxant activity, at least at the highest concentrations assessed. Surprisingly, under this experimental condition, **5** caused a dramatic concentration-dependent increase of phenylephrine-induced tone.

### 2.4. Biophysical and Pharmacological Analysis of the Effect of ***7***, ***8***, and ***9*** on Ca_V_1.2 Channels

The voltage dependence of **7**, **8**, and **9** inhibition of I_Ba1.2_ was further investigated by analyzing the steady-state inactivation and activation curves. The steady-state activation curves, calculated from the current–voltage relationships shown in Figure 3, were fitted with the Boltzmann equation.

**Figure 5:**
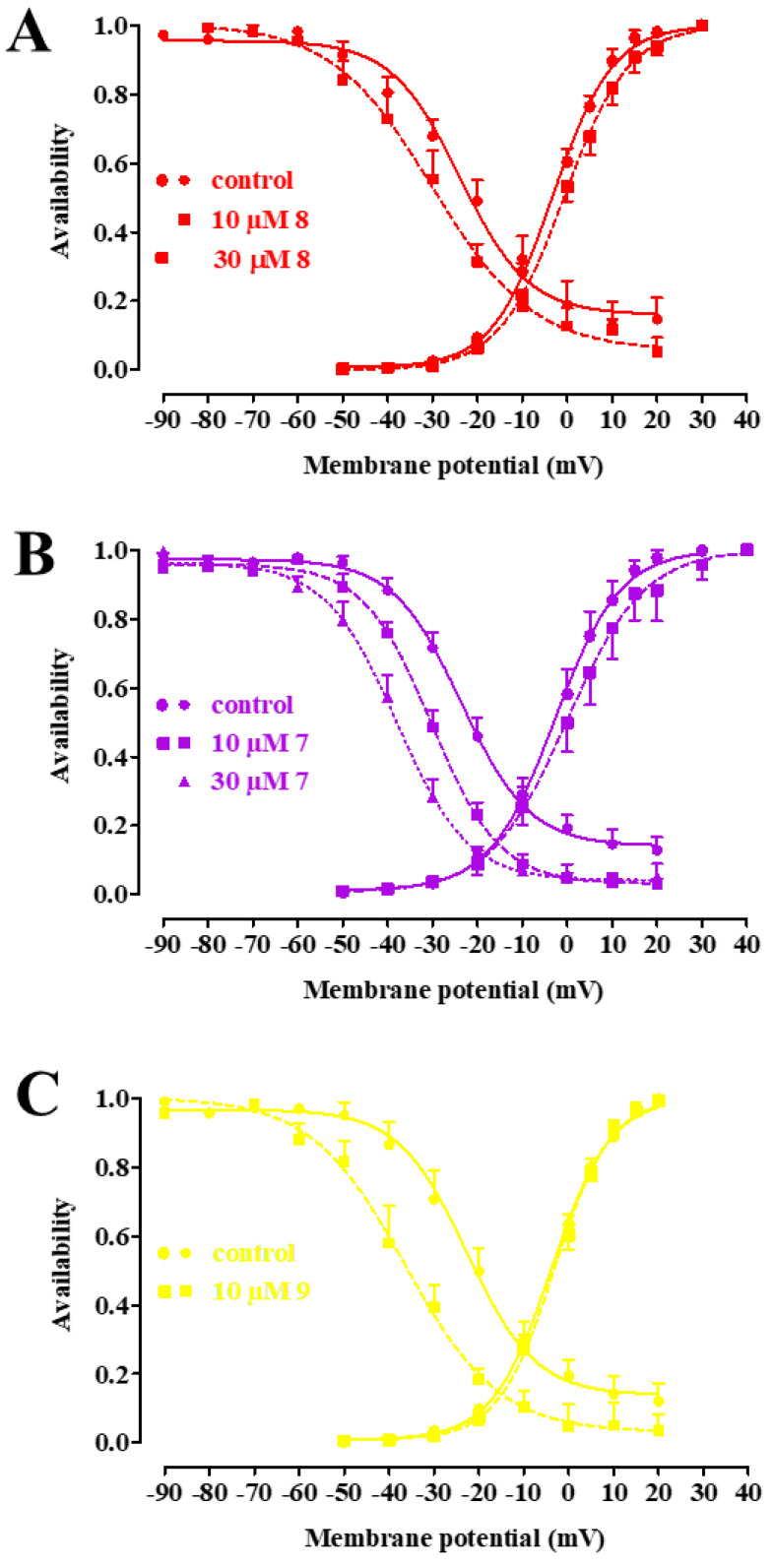
Effect of **8**, **7**, and **9** on the voltage dependence of Ca_v_1.2 channel activation and inactivation in single rat tail artery myocytes. Steady-state inactivation curves, recorded from a V_h_ of −80 mV in the absence (control) or presence of various concentrations of (**A**) **8**, (**B**) **7**, and (**C**) **9**, were fitted to the Boltzmann equation. Peak current values were used. The current measured during the test pulse is plotted against membrane potential and expressed as availability. Steady-state activation curves were obtained from the current–voltage relationships of Figure 3 and fitted to the Boltzmann equation. Data points are mean ± s.e.m. (*n* = 5–6).

The three compounds significantly shifted the steady-state inactivation curve to more negative potentials, with **7** also doing so in a concentration-dependent manner (Figure 5A–C), and reduced the 50% inactivation potential (Table 2), but only **7** significantly increased the 50% activation potential. The slope factor (which describes the steepness of the curve, with a larger value denoting a shallow curve) of both activation and inactivation curves remained unaffected.

**Figure 6:**
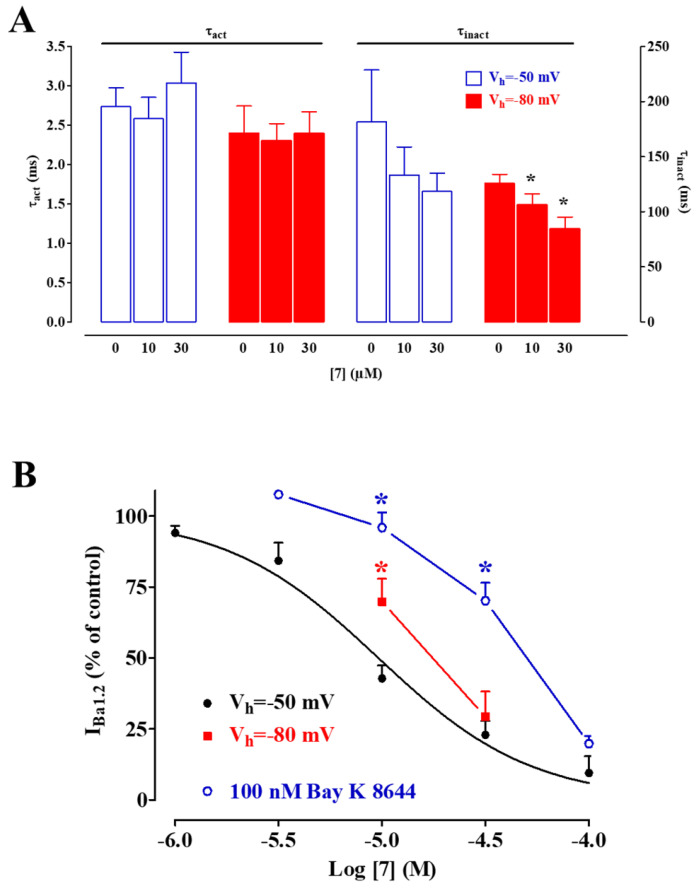
Biophysical and pharmacological features of **7**-induced blockade of single rat tail artery myocyte Ca_V_1.2 channels. (**A**) Effect of **7** on I_Ba1.2_ kinetics. Time constant for activation (τ_act_) and inactivation (τ_inact_) measured in the absence or presence of different concentrations of **7** from a V_h_ of either −50 mV or −80 mV. Columns represent the mean ± s.e.m. (*n* = 5–7). * *p* < 0.05 vs. control, repeated measures ANOVA and Dunnett’s post-hoc test. (**B**) Effect of membrane potential and Bay K 8644 on I_Ba1.2_ inhibition induced by various concentrations of **7**. I_Ba1.2_, elicited by 250-ms clamp pulses to 10 mV from a V_h_ of either −50 mV (in the absence or presence of 100 nM Bay K 8644) or −80 mV, measured after the addition of cumulative concentrations of **7**. The concentration–response curve to **7** at a V_h_ of −50 mV is taken from Figure 2C. On the ordinate scale, the current amplitude is reported as a percentage of the value recorded just before the addition of the first concentration of the compound. Data points represent the mean ± s.e.m. (*n* = 5–7). * *p* < 0.05 vs. control, repeated measures ANOVA and Dunnett post-hoc test.

The shift of both the activation and inactivation curve caused by 10 µM **7** led to a marked reduction in the Ba^2+^ window current, which peaked at about −15 mV (with a relative amplitude of 0.021), compared with that observed under the control conditions, which peaked at −10 mV (relative amplitude 0.078). I_Ba1.2_ evoked at 10 mV from a V_h_ of −50 mV activated and then declined with time courses that could be fitted by a mono-exponential function. Moreover, **7** significantly accelerated, in a concentration-dependent manner, the τ of inactivation (though significance was reached only at a V_h_ of −80 mV), without affecting that of activation (Figure 6A). This series of experiments was carried out to investigate whether the dihydropyridine binding site on the channel protein was involved in the Ca^2+^ antagonistic activity of **7** and to evaluate the voltage-dependence of the binding. As shown in Figure 6B, when the membrane potential was shifted from −50 mV to −80 mV, the inhibitory activity of **7** significantly decreased. A further decrease was observed when the potential functional interaction of **7** and the Ca_V_1.2 channel stimulator Bay K 8644 was assessed. In myocytes challenged with 100 nM Bay K 8644, I_Ba1.2_ increased to 447 ± 28% of the control (*n* = 5). As shown in Figure 6B, pretreatment with Bay K 8644 caused a significant rightward shift of the **7** concentration–response curve.

### 2.5. Effect of ***7*** on K_Ca_1.1 Channels

The functional experiments indicated that **7** behaves as a K^+^ channel opener, being more active with a 25 mM KCl- as compared to 60 mM KCl-induced contraction. Therefore, **7** was also assessed for its effect on I_KCa1.1_. Under the conditions used in the present experiments, the outward current mostly consisted of iberiotoxin-sensitive I_KCa1.1_ [26]. 

**Figure 7:**
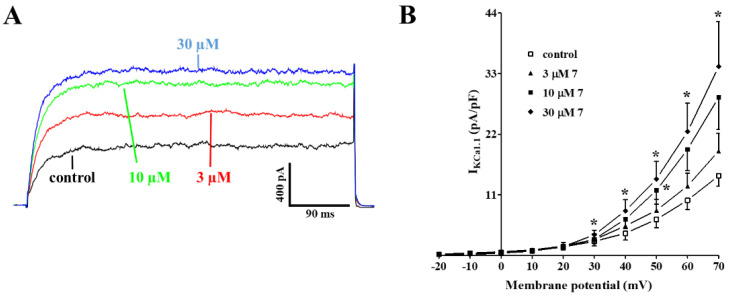
Effects of **7** on K_Ca_1.1 channels. (**A**) Average traces (recorded from five cells) of I_KCa1.1_ measured in tail artery myocytes, in the absence (control) or presence of various concentrations of **7**. I_KCa1.1_ was elicited with a clamp pulse to 70 mV from a V_h_ of −40 mV, delivered every 10 s. (**B**) Effect of **7** on the current–voltage relationship. On the ordinate scale, I_KCa1.1_ amplitude is reported in pA/pF. Data points are mean ± s.e.m. (*n* = 5). * *p* < 0.05 vs. control, repeated measures ANOVA and Dunnett post-hoc test.

**Figure 8:**
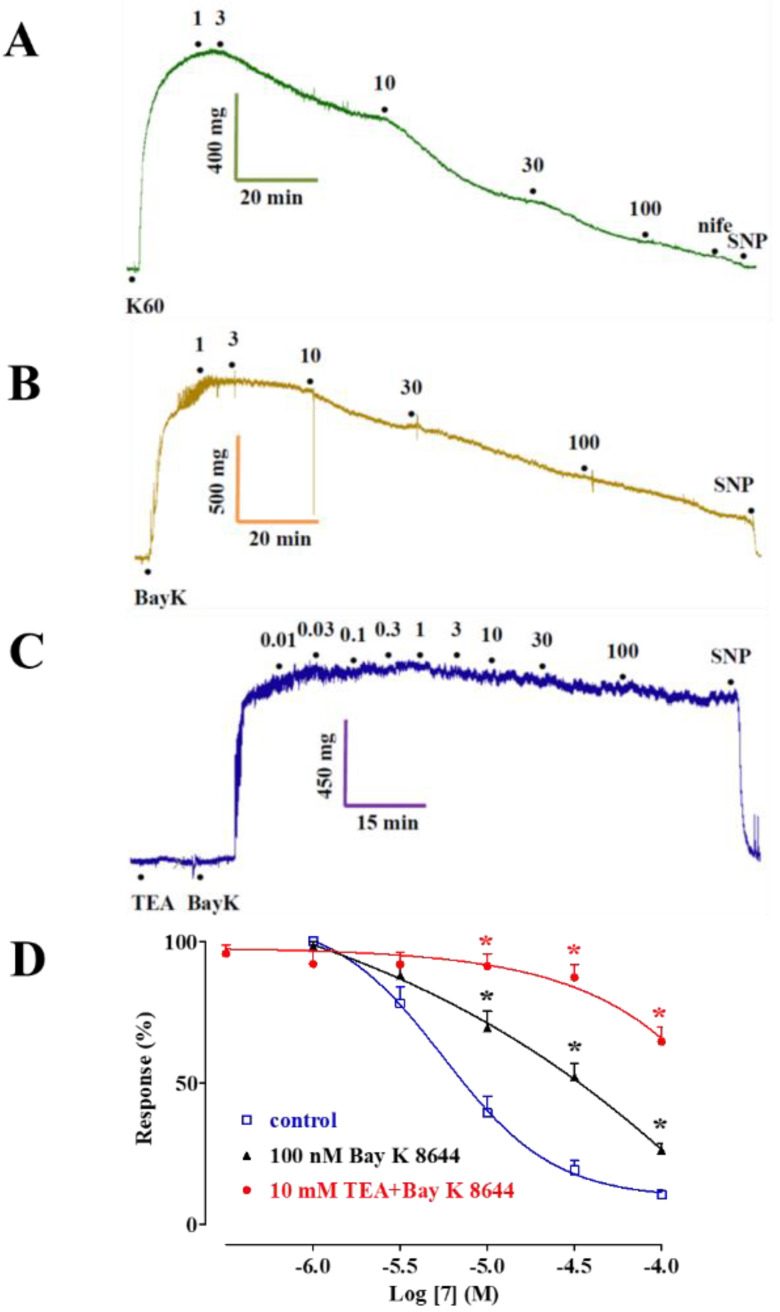
Effect of Bay K 8644 and TEA on the vasorelaxant activity of **7**. (**A**) Control rings were stimulated by 60 mM KCl, or rings were pre-incubated with (**B**) 100 nM Bay K 8644 alone or (**C**) Bay K 8644 plus 10 mM TEA for 10 min, and were stimulated using 10–15 mM KCl. After a steady contraction was obtained, **7** was added cumulatively. (**D**) Concentration–response curve to **7**. In the ordinate scale, the response is reported as a percentage of the initial tension. Data points represent the mean ± s.e.m. (*n* = 6–7). * *p* < 0.05 vs. control, repeated measures ANOVA and Dunnett post-hoc test vs. control.

Figure 7A shows the traces of I_KCa1.1_ elicited with clamp pulses to 70 mV from a V_h_ of −40 mV, under control conditions and after the cumulative addition of 3 µM, 10 µM, and 30 µM **7**, which caused a significant concentration-dependent stimulation of the current, observed in a range of membrane potential 30–70 mV (Figure 7B). The electrophysiology data pointed to **7** as a Ca_V_1.2 channel blocker and a K_Ca_1.1 channel stimulator. To confirm this evidence at the tissue level, aorta rings were pre-contracted using 60 mM KCl in the presence of either the Ca_V_1.2 channel stimulator Bay K 8644 (100 nM) or Bay K 8644 plus the K^+^ channel blocker tetraethylammonium chloride (TEA, 10 mM, a concentration known to block most of K^+^ channels [27]. As shown in Figure 8, the concentration–response curve to **7** was significantly shifted to the right when Bay K 8644 was present in the organ bath. A further, significant shift was observed in the presence of both Bay K 8644 and TEA.

### 2.6. Docking and Classical Molecular Dynamics Simulations

**Figure 9:**
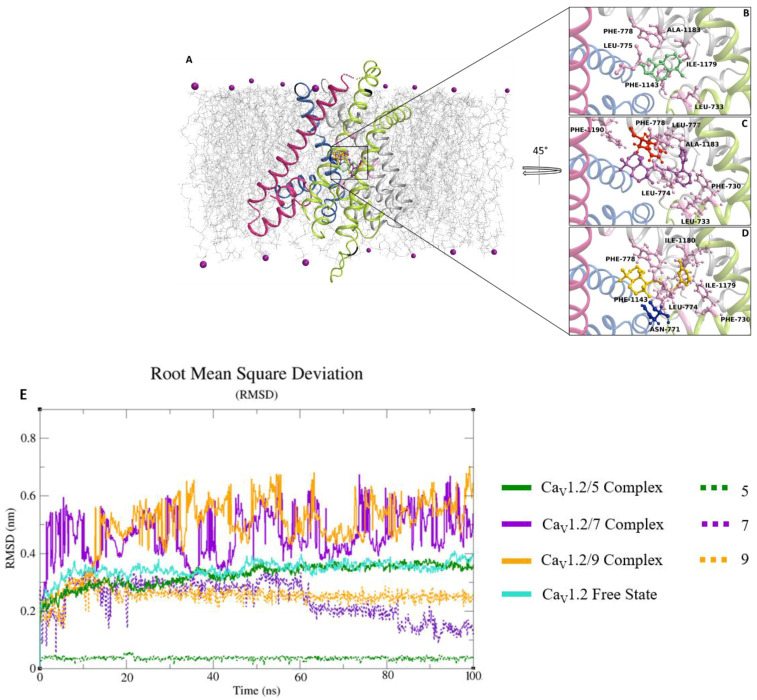
Root mean square deviation (RMSD) profiles of compounds in complex with the Ca_V_1.2 channel, and overview of the channel binding pocket docked with **5**, **7**, and **9**. (**A**–**D**) The Ca_V_1.2 channel 3D structure is depicted in a multicolor cartoon. The bilayer is depicted as grey lines, while some phospholipid heads are reported in purple spheres. Interaction network of (**B**) **5,** (**C**) **7** and, (**D**) **9** in complex with the Ca_V_1.2 binding residues after the docking simulation. The residues involved in π-stacking and hydrogen bonds are reported as red and blue ball/sticks, respectively. The hydrogen atoms are hidden for clarity. (**E**) RMSD profiles of Ca_V_1.2 channel backbone in complex with **5**, **7**, and **9**. The RMSD trends are represented as colored lines (see legend). The RMSD (nm) and time (ns) values of the MD run are reported on the Y- and X- axis, respectively.

The best-docked pose of **7**, **9**, and **5** on the homology model of the *Rattus norvegicus* Ca_V_1.2 channel α_1C_ subunit exhibited Gibbs free-energy values (ΔG) of −6.2 kcal/mol, −7 kcal/mol, and −6.3 kcal/mol, respectively. The compounds shared the same binding region, close to the central pore. The interaction network analyzed by the P.L.I.P. tool demonstrated that **5** was only able to form hydrophobic interactions with Leu-733, Leu-775, Phe-778, Ile-1179, Ala-1183, and Phe-1143 (Figure 9A). Whereas, **9** triggered hydrophobic interactions with Phe-730, Leu-733, Leu-774, Leu-777, Phe-778, Ala-1183, and Phe-1190, and a π-stacking with Phe-778 (Figure 9B). In addition, **7** exhibited hydrophobic interactions with Phe-730, Leu-774, Phe-778, Phe-1143, Ile-1179, and Phe-1180 (Figure 9C) and a hydrogen bond with Asn-771. The root mean square deviation (RMSD) analyzed on the backbone of the Ca_V_1.2 channel in complex with **5**, **7**, and **9** showed stable profiles during the molecular dynamics run, indicating a good structural integrity of the channel (Figure 9D).

**Figure 10:**
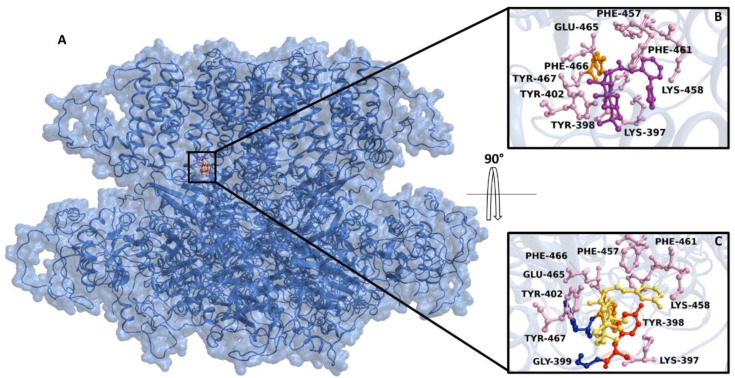
Overview of the K_Ca_1.1 channel binding pocket in complex with **7** and **9**. (**A**) The K_Ca_1.1 channel 3D structure is depicted in the cyan transparency surface and cartoon. Interaction network of (**B**) **7** and (**C**) **9** in complex with the K_Ca_1.1 channel binding residues. The residues involved in π-stacking, hydrogen bond, and salt bridge, are reported as red, blue, and orange ball/sticks, respectively. The hydrogen atoms are hidden for clarity.

Interestingly, the Ca_V_1.2 channel in a free state and the Ca_V_1.2 channel/**5** complex showed a similar RMSD trend, differently from Ca_V_1.2 channel/**7** and **9** complexes, which exhibited a comparable RMSD. Similarly, the compounds showed a good stability, revealing RMSD values between 0.05 nm and 0.25 nm (Figure 9D), confirming the reliability of the initial docking binding pose. The non-bonded interaction energy of the target in complex with **7**, **9**, and **10** showed values of -195.3 ± 10.3 kJ/mol (−46.7 ± 2.4 kcal/mol), −137.2 ± 3.7 kJ/mol (−32.7 ± 0.35 kcal/mol), and −127 ± 3 kJ/mol (−30.3 ± 0.7 kcal/mol), respectively. In silico results showed that **7** and **9** were able to bind spontaneously in the same region of the K_Ca_1.1 channel (Figure 10A) showing Gibbs free-energy values (ΔG) of −5.9 kcal/mol and −4.4 kcal/mol, respectively. The interaction network analyses demonstrated that **7** triggered four hydrophobic interactions with Lys-397, Tyr-398, Tyr-402, Phe-457, Lys-458, Phe-461, Glu-465, and Tyr-467, and a salt bridge with Phe-466 (Figure 10B), whereas **9** was involved in four hydrophobic interactions with Lys-397, Tyr-398, Tyr-402, Phe-457, Lys-458, and Phe-461, two hydrogen bonds with Gly-399 and Tyr-467, and a π-stacking with Tyr-398 (Figure 10C). 

### 2.7. Effect of ***7*** on Langendorff Perfused Rat Heart

**Figure 11:**
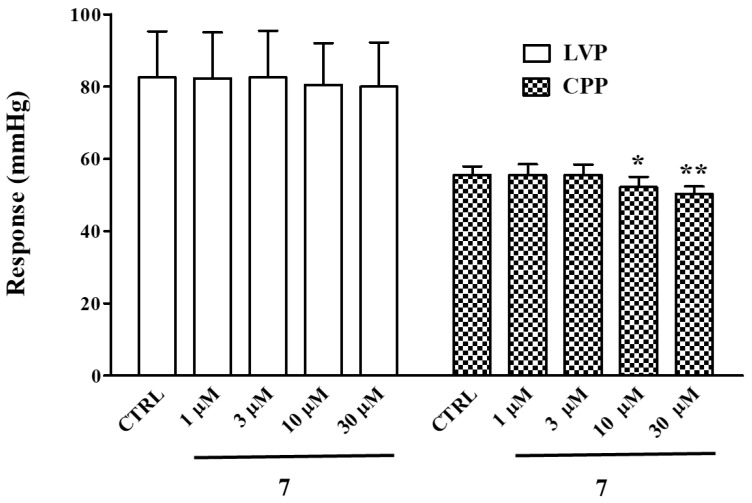
Effects of compound **7** on LVP and CPP in Langendorff-perfused rat hearts. Concentration–effect relationship of compound **7** on LVP and CPP. On the ordinate scale, the response is reported as mmHg. Each value represents mean ± s.e.m. (*n* = 5). * *p* < 0.05, ** *p* < 0.01 vs. CTRL, repeated measures ANOVA and Dunnett’s post-test.

**Figure 12:**
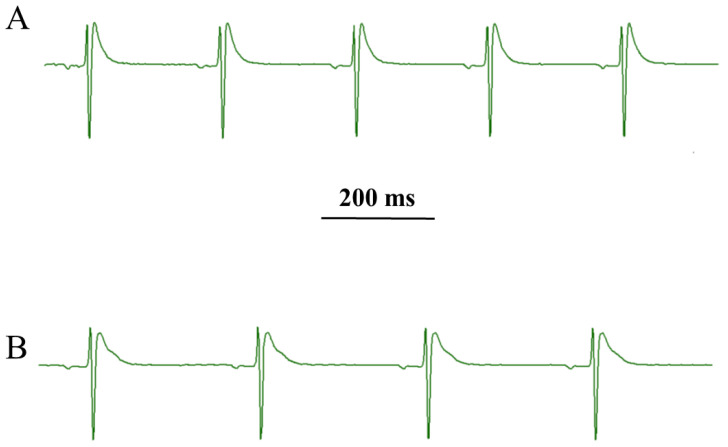
Original ECG trace recorded under (**A**) control conditions and (**B**) in the presence of 30 µM **7**.

Under control conditions, left ventricular pressure (LVP) and coronary perfusion pressure (CPP) values of 82.58 ± 12.74 and 55.58 ± 2.33 mmHg (*n* = 5), respectively, were obtained. Compound **7** significantly decreased CPP, leaving LVP unaltered (Figure 11). At the maximum concentration tested (30 µM), HR was significantly reduced, while both RR and PQ intervals were augmented (Table 3, Figure 12). However, QRS and QTc values did not vary over the drug concentration range tested.

## 3. Discussion

Several pieces of evidence support the successful strategy pursued here to improve the vasorelaxant activity of **5**, which gave rise to **7**:

1. chemical modification of **5** generated effective vasodilating agents that were more potent Ca_V_1.2 channel blockers than the parent compound;
2. their Ca^2+^ antagonism seems to be related to the stabilization of the Ca_V_1.2 channel inactivated state;
3. a functional endothelium is not crucial for, and in some cases counteracts, their activity, though to a lesser extent as compared to **5**;
4. **7**, besides blocking Ca_V_1.2 channels, is also capable of stimulating the K_Ca_1.1 channels;
5. **7** is a negative chronotropic and coronary vasodilating agent.

From a chemical point of view, **5** is characterized by a central labdane nucleus and a condensed five-members-lactone, whose integrity seems crucial for many of its biological activities. However, its opening along with the introduction of an amide/ether bridge gave rise to different small molecules endowed with vasorelaxant activity, with **7** being the most potent, even over the parent compound **5**. Compounds **8** and **9** resulted as effective, though less than **7**, whereas **10** failed. The structural features of the derivatives may explain this different pharmacological activity. Compound **10** has an ether bridge, while **7**, **8**, and **9** present an amide, i.e., an additional H-donor group and a carbonyl moiety. Furthermore, the opening of the five-member lactone gave rise to a labdane head linked to an aryl tail through an amide bridge 3 (**7**), 4 (**8**), and 5 (**9**) atoms long. The most promising was identified in compound **7** (3-atoms long), which was demonstrated to behave as a potent vasodilating agent. From a pharmacological point of view, all the derivatives but **10** reverted the high KCl-induced contraction (which is essentially due to extracellular Ca^2+^ influx through Ca_V_1.2 channels), with a potency and an efficacy greater than those of the parent compound. The patch–clamp experiments confirmed the hypothesis that these derivatives are Ca_V_1.2 channel blockers. Noticeably, their IC_50_ values were comparable to those of drugs currently used in therapy, e.g., verapamil and diltiazem, as measured with the same model system [28]. Furthermore, the current–voltage relationship analysis demonstrated that the response of the channel to changes in membrane potential was not affected either by **5** or by its derivatives. The experiments performed in the presence of the Ca_V_1.2 channel agonist Bay K 8644, on the one hand, further strengthened the hypothesis that the most potent compound, i.e., **7**, is a channel blocker, because its efficacy was markedly reduced under this experimental condition. On the other hand, they suggested that the dihydropyridine binding site or, at least, a site allosterically linked, might be targeted by the compound. Interesting clues arose from the biophysical analysis of the effect of **7** on Ca_V_1.2 channels. The molecule, similarly to **8** and **9**, shifted the steady-state inactivation curve to more negative potentials, indicating that this scaffold is capable of stabilizing the channel in the inactivated state. Consistent with this hypothesis, **7** speeded up the inactivation kinetics of the I_Ba1.2_ (i.e., accelerated the transition from the open to the inactivated state) and reduced its efficacy when the membrane potential was held at a more hyperpolarized value, i.e., when a large number of channels were in the resting, closed state. On the contrary, neither the activation kinetics nor the steady-state activation curve was affected by **7**, suggesting that the molecule does not modify the voltage sensitivity of the channel and the transition from the resting to the open state.

In silico results suggested that **7**, **9**, and **10** bound spontaneously to a Ca_V_1.2 channel binding pocket, where other blockers were located [29], with high stability and affinity, triggering a hydrophobic and polar interaction network. However, the functional results showed that only **7** and **9** were able to significantly counteract vessel contraction after Ca_V_1.2 channel opening. The fact that **7** and **9** were the only compounds able to form a π-stacking and a hydrogen bond with Phe-778 and Asn-771 (corresponding to Phe-656 and Asn-649 of the rabbit Ca_V_1.1 channel), respectively, two key residues both for the binding of known Ca_V_1.2 channel blockers and for channel inhibition, might explain this apparent discrepancy. However, only mutagenesis experiments can support and strengthen this hypothesis. Notably, the RMSD profiles of the Ca_V_1.2 channel in the free state and in complex with **5** were superimposable, suggesting that **5**, contrary to **7** and **9**, did not alter the structural stability of the channel and likely its structural dynamics, whereas the complex with **7** and **9** showed higher RMSD values, as compared to the free state. Altogether, both the structural and energy evidence provided by the cMD analysis were consistent with the in vitro results, indicating the ability of **7** and **9** to impact the biological function more markedly than **5**.

Other pathways beyond Ca_V_1.2 channels are key regulators of vascular smooth muscle tone, namely K_Ca_1.1 channels, intracellular Ca^2+^ stores, as well as factors released by the endothelium. The opening of K_Ca_1.1 channels caused membrane depolarization and the subsequent closure of Ca_V_1.2 channels. This effect, however, is masked when the K^+^ gradient across the membrane is reduced by, for example, the addition of high KCl concentration to the physiological solution. Therefore, the vasorelaxant activity of K_Ca_1.1 channel openers can only be observed when the vascular smooth muscle contraction is brought about by a moderate increase in extracellular K^+^ concentration. Under this experimental condition, the spasmodic potency of **7**, **8**, and **9** increased significantly, providing substantial evidence that these molecules might open K^+^ channels, thus triggering membrane hyperpolarization. Indeed, **7** stimulated I_KCa1.1_ in a concentration-dependent manner, thus proving this hypothesis.

Gessner et al. [30], through experimental mutagenesis, showed that the S6/RCK linker in the K_Ca_1.1 channel is crucial for both channel activation and binding of modulators. In silico results showed that **7** and **9** bound close to this region, exhibiting a high binding free energy. This observation, however, needs to be validated through experimental mutagenesis. Unfortunately, due to the low cover value of our target against the template in some protein regions and the low confidence in their secondary structure prediction, cMD could not be performed, thus limiting a thorough investigation of the derivative binding mode.

In aorta rings pre-contracted with phenylephrine, the vasoactivity of **5** and its derivatives was low. The selective stimulation of vascular smooth muscle α_1_ receptors caused an increase in the cytoplasmic Ca^2+^ concentration that triggered vessel contraction. Ca^2+^ is released by the IP3-sensitive intracellular stores and enters the extracellular space through voltage-dependent, receptor-, and store-operated Ca^2+^ channels [31]. The weak efficacy of derivatives toward phenylephrine-induced contraction is consistent with the hypothesis that they are selective Ca_V_1.2 channels blockers and, therefore, less effective on phenylephrine- as compared to high K^+^-induced contraction. In the former, in fact, Ca_V_1.2 channels play a minor role. 

Surprisingly, when the endothelium was present, the vasorelaxant activity of **7**, **8**, and **9** vanished, while that of **5** converted to a marked contraction. It is hypothesized that the compounds stimulate the release of contracting factors from the endothelium, e.g., prostanoids or endothelin-1. Noticeably, the derivatization strategy prevented the endothelium-dependent vasoconstricting activity of **5** (which might give rise to harmful clinical effects), once more proving successful.

Sometimes, whole-cell patch-clamp data, obtained in single myocytes undergoing extensive dialysis of the cytoplasm, cannot be reproduced in a whole tissue, where the myocyte is intact and is a brick of a complex physiological environment. However, the results presented here clearly demonstrate that in the intact tissue, Ca_V_1.2 channel blockade and K_Ca_1.1 channel stimulation underpin **7**-induced vasorelaxation. In fact, its spasmolytic activity was markedly reduced in tissues pre-treated with Bay K 8644 and, moreover, when also TEA was present.

Given the key role played by these channels, in particular the Cav1.2 channel, in the electrical and mechanical activity of the heart, the cardiac effects of **7** were investigated in the Langendorff perfused rat heart. Beyond its effects on the excitation–contraction coupling of vascular smooth muscle, **7** exhibited a negative chronotropic and coronarodilating activity, and prolonged the cardiac cycle length, as well the atrioventricular conduction time. These activities likely reflected its Ca^2+^ antagonist property. In fact, drugs capable of inhibiting Ca_V_1.2 channel current reduce HR and prolong conduction and refractoriness of the atrioventricular node [13,32,33].

In conclusion, a sclareolide (**5**) scaffold represents a valuable starting point for the development of bifunctional vasorelaxant agents endowed with negative chronotropic properties and targeting key pathways involved in the pathophysiology of hypertension and ischemic cardiomyopathy.

## 4. Materials and Methods

### 4.1. Chemicals

Semi-synthetic tools **6**–**10** were prepared as previously described [25]. The chemicals used were acetylcholine, BSA, collagenase (type XI), nifedipine, phenylephrine, (S)-(-)-Bay K 8644, taurine, TEA, and soybean trypsin inhibitor (Sigma Chimica, Milan, Italy); sodium nitroprusside (Riedel-De Haen AG, Seelze Hannover, Germany). All other substances were of analytical grade and used without further purification. Phenylephrine was solubilized in 0.1 M HCl. Nifedipine and Bay K 8644 were dissolved directly in ethanol, with **5** and its derivatives **7**–**10** in DMSO. Control experiments confirmed that no response was observed in vascular preparations when either DMSO or ethanol, at the final concentration used in the above dilutions (0.1%, *v/v*), were added alone (data not shown).

### 4.2. Animal Care Statement

All the study procedures were in strict accordance with the European Union Guidelines for the Care and the Use of Laboratory Animals (European Union Directive 2010/63/EU) and approved by the Animal Care and Ethics Committee of the University of Siena and the Italian Department of Health (7DF19.N.TBT). The heart, abdominal aorta, and tail main artery were isolated from male Wistar rats (250–350 g, Charles River Italia, Calco, Italy) maintained in an animal house facility at 25 ± 1 °C and 12:12 h dark–light cycle with access to standard chow diet and water ad libitum, as previously described [28,34,35].

### 4.3. Aorta Ring Preparation

The abdominal aorta was gently cleaned of adipose and connective tissues and cut into 3-mm wide rings. These were mounted in organ baths between two parallel, L-shaped, stainless-steel hooks, with one fixed in place and the other connected to an isometric transducer. Rings were allowed to equilibrate for 60 min in modified Krebs-Henseleit solution (composition in mM: 118 NaCl, 4.75 KCl, 1.19 KH_2_PO_4_, 1.19 MgSO_4_, 25 NaHCO_3_, 11.5 glucose, 2.5 CaCl_2_, gassed with a 95% O_2–_5% CO_2_ gas mixture to create a pH of 7.4) under a passive tension of 1 g. During this equilibration period, the solution was changed every 15 min. Isometric tension was recorded using a digital PowerLab data acquisition system (PowerLab 8/30; ADInstruments). Ring viability was assessed by recording the response to 0.3 µM phenylephrine and 60 mM KCl. Where needed, the endothelium was removed by gently rubbing the lumen of the ring with a forceps tip. This procedure was validated by adding 10 µM acetylcholine at the plateau of phenylephrine-induced contraction; a relaxation greater than 70% or less than 10% denoted the presence or absence of functional endothelium, respectively [36].

### 4.4. Effect of Sclareolide and Its Derivatives **7**–**10** on Phenylephrine- or High KCl-Induced Contraction

Aorta rings were pre-contracted pharmaco-mechanically using 0.3 µM phenylephrine and electromechanically using 25–30 mM or 60 mM KCl. Once the contraction reached a plateau, the compound was added cumulatively into the organ bath [37]. At the end of the concentration-response curve, 100 µM sodium nitroprusside alone (phenylephrine-induced contraction) or 1 µM nifedipine followed by sodium nitroprusside (K^+^-induced contraction) were added to test the functional integrity of smooth muscle. The response was calculated as the percentage of phenylephrine- or KCl-induced contraction (taken as 100%).

### 4.5. Cell Isolation Procedure

The tail main artery was dissected free of its connective tissue, and smooth muscle cells were freshly isolated under the following conditions. A 5-mm long piece of artery was incubated at 37 °C for 40–45 min in 2 mL of 0.1 mM Ca^2+^ external solution (consisting of (in mM): 130 NaCl, 5.6 KCl, 10 HEPES, 20 glucose, 1.2 MgCl_2_, and 5 Na-pyruvate; pH 7.4) containing 20 mM taurine, which replaced an equimolar amount of NaCl, 1.35 mg/mL collagenase (type XI), 1 mg/mL soybean trypsin inhibitor, and 1 mg/mL BSA. This solution was gently bubbled and stirred with a 95% O_2–_5% CO_2_ gas mixture, as previously described [28]. Cells stored in 0.05 mM Ca^2+^ external solution containing 20 mM taurine and 0.5 mg/mL BSA at 4 °C under normal air were used for experiments within two days after isolation [28].

### 4.6. Whole-Cell Patch-Clamp Recordings

An Axopatch 200B patch-clamp amplifier (Molecular Devices Corporation, Sunnyvale, CA, USA) was used to generate and apply voltage pulses to the clamped cells and record the corresponding membrane currents. Recording electrodes were pulled from borosilicate glass capillaries (WPI, Berlin, Germany) and fire-polished to obtain a pipette resistance of 2–4 MΩ, when filled with an internal solution. At the beginning of each experiment, the junction potential between the pipette and bath solution was electronically adjusted to zero. After compensation for whole-cell capacitance and series resistance (between 70% and 75%), current signals were low-pass filtered at 1 kHz and digitized at 3 kHz, before being stored on a computer hard disk. Electrophysiological responses were tested at room temperature (20–22 °C) [38].

### 4.7. Ba^2+^ Current through Ca_V_1.2 Channel (I_Ba1.2_) Recordings

Cells were continuously superfused with an external solution containing 0.1 mM Ca^2+^ and 30 mM TEA using a peristaltic pump (LKB 2132, Bromma, Sweden) at a flow rate of 400 µL/min. The conventional whole-cell patch-clamp method was employed to voltage-clamp smooth muscle cells. The internal solution consisted of (in mM) 100 CsCl, 10 HEPES, 11 EGTA, 2 MgCl_2_, 1 CaCl_2_ (pCa 8.4), 5 Na pyruvate, 5 succinic acid, 5 oxaloacetic acid, 3 Na_2_ATP, and 5 phosphocreatine; pH was adjusted to 7.4 with CsOH. I_Ba1.2_ was recorded in an external solution containing 30 mM TEA and 5 mM Ba^2+^. The current was elicited with 250-ms clamp pulses (0.067 Hz) to 10 mV, from a V_h_ of −50 mV. Data were collected once the current amplitude had been stabilized (usually 7–10 min after the whole-cell configuration had been obtained). Under these conditions, the current did not run down during the following 40 min [39]. 

Steady-state activation curves were derived from the current–voltage relationships. Conductance (G) was calculated from the equation G = I_Ba1.2_/(E_m_ − E_rev_), where: I_Ba1.2_ is the peak current elicited by depolarizing test pulses between −50 mV and 40 mV from a V_h_ of −50 mV; E_m_ is the membrane potential; and E_rev_ is the reversal potential (estimated from the extrapolated current-voltage curves in Figure 2). G_max_ is the maximal Ba^2+^ conductance (calculated at potentials ≤40 mV). The G/G_max_ ratio was plotted against the membrane potential and fitted to the Boltzmann equation [40]. Steady-state inactivation curves were obtained using a double-pulse protocol. Once various levels of the conditioning potential had been applied for 5 s, followed by a short (5-ms) return to the V_h_ of −80 mV, a test pulse (250 ms) to 10 mV was delivered to evoke the current. The delay between the conditioning potential and the test pulse allowed the full or near-complete deactivation of the channels, simultaneously avoiding partial recovery from inactivation. K^+^ currents were blocked with 30 mM TEA in the external solution and Cs^+^ in the internal solution. Current values were corrected for leakage and residual outward currents using 10 µM nifedipine, which completely blocked I_Ba1.2_. The osmolarity of the 30 mM TEA- and 5 mM Ba^2+^-containing external solution (320 mosmol, adjusted with NaCl if required) and that of the internal solution (290 mosmol) were measured with an osmometer (Osmostat OM 6020, Menarini Diagnostics, Florence, Italy).

### 4.8. K^+^ Current through K_Ca1.1_ Channel (I_KCa1.1_) Recordings

K^+^ current through the K_Ca1.1_ channel (I_KCa1.1_) (registration period 500 ms) was measured over a range of test potentials, from −20 mV to 70 mV from a V_h_ of −40 mV. This V_h_ limited the contribution of voltage-dependent K^+^ channels to the overall whole-cell current. Data were collected once the current amplitude had been stabilized (usually 6–10 min after the whole-cell configuration had been obtained). I_KCa1.1_ did not run down during the following 20–30 min under the present experimental conditions [41]. External solution for I_KCa1.1_ recordings contained (in mM): 145 NaCl, 6 KCl, 10 glucose, 10 HEPES, 5 Na-pyruvate, 1.2 MgCl_2_, 0.1 CaCl_2_, 0.003 nicardipine (pH 7.4). The internal solution contained (in mM): 90 KCl, 10 NaCl, 10 HEPES, 10 EGTA, 1 MgCl_2_, 6.41 CaCl_2_ (pCa 7.0; pH 7.4). The osmolarity of the external and internal solutions was 310 mosmol and 265 mosmol, respectively. The current–voltage relationships were calculated based on the values recorded during each test pulse (leakage corrected offline). I_KCa1.1_ was isolated from other currents, as well as corrected for leakage using 1 mM TEA, a specific blocker of K_Ca1.1_ channels [27].

### 4.9. Isolated Rat Heart Preparation and Perfusion

Spontaneously beating heart was retrogradely perfused via the aorta at a constant perfusion flow in a Langendorff apparatus (Radnoti, Dublin, Ireland) with a physiological salt solution containing (mM) NaCl 118, KCl 4.7, CaCl_2_ 2.5, MgSO_4_ 1.2, NaHCO_3_ 25, KH_2_PO_4_ 1.2, glucose 11.5, Na pyruvate 2, and EDTA 0.5, and bubbled with a 95% O_2_−5% CO_2_ gas mixture (pH 7.4), and kept at 37 °C, as described elsewhere [42,43,44]. Hearts were allowed to equilibrate for at least 20 min before **7** exposure. Heart contractility was measured as left ventricle developed pressure (LVDP) using a deionized water-filled latex balloon, connected to a pressure transducer (BLPR, WPI, Berlin, Germany) and inserted into the left ventricular cavity through the mitral valve. A pressure transducer (BLPR, WPI, Berlin, Germany) was placed in the inflow line, to record coronary perfusion pressure (CPP), a measure of coronary vascular resistance [45]. Two steel electrodes, one positioned on the left atrium and the other on the apex of the heart, were used to record the surface electrocardiogram. Heart rate (HR) and RR (cycle length), PQ (atrioventricular conduction time), QRS (intraventricular conduction time), and QT (ventricular depolarization and repolarization) intervals were measured [46]. The digital PowerLab data acquisition system (PowerLab 8/30; ADInstruments, Castle Hill, Australia) and Chart Pro for Windows software v. 7.3.7 (PowerLab; ADInstruments, Castle Hill, Australia) were used to record and analyze LVP, CPP, and ECG. As the QT interval is affected by HR changes (e.g., it shortens when HR increases), Bazett's formula normalized to average rat RR (QTc = QT/(RR/f)1/2) [46] was routinely used to correct it, thus avoiding confounding effects. In this study, “f” (the normalization factor according to the basal RR duration) was 231.4 ms, as this was the average cardiac cycle length. 

### 4.10. Statistical Analysis

Analysis of data was accomplished using LabChart 7.3.7 Pro (PowerLab; ADInstruments), pClamp 8, and GraphPad Prism 5.04 (GraphPad Software Inc.). Data are reported as mean ± s.e.m.; *n* is the number of rings or cells analyzed (indicated in parentheses), isolated from at least three animals. Statistical analysis and significance, as measured by one-way or repeated measures ANOVA (followed by Dunnett post-hoc test) or Student’s t test for paired samples (two-tailed), were obtained using GraphPad Prism 5.04 (GraphPad Software Inc.). In all comparisons, *p* < 0.05 was considered significant. The pharmacological response to drugs, described in terms of potency (IC_50_ value, i.e., the drug concentration that caused a decrease of response equal to 50% of the maximum value) and efficacy (E_max_, i.e., the maximum response achieved with the highest concentration tested), was obtained by nonlinear regression analysis.

### 4.11. In Silico Methods: Structural Resource

The *Rattus norvegicus* Ca_V_1.2 channel subunit α_1C_ 3D structure was achieved with a homology modelling procedure, as described by Trezza et al. [47]. The primary structure of *Rattus norvegicus* K_Ca_1.1 channel, downloaded by Uniprot Database (UniProt ID - Q62976 -), was used as a query sequence for a multiple sequences alignment (MSA) carried out using Clustal Omega, implemented in PyMOD3.0 [48], and choosing Protein Data Bank (pdb) as the Database; all algorithm parameters were used as default. As evidenced by the MSA results, the Cryo-EM structure of the Ca^2+^-bound hsSlo1-beta4 channel complex (PDB code: 6V22) was the best template [49], showing a cover and identity of 90.2% and 99.4%, respectively. Then, 6V22 was identified as a template to rebuild the 3D structure of the *Rattus norvegicus* K_Ca_1.1 channel, using the Modeller tool implemented in PyMOD3.0. The validity of the 3D structure was assessed using Ramachandran plot and PROCHECK analyses, as previously described [50]. The 3D structures of compounds **5**, **7**, and **9** were drawn with the ChemDraw software. Compounds were sketched and then prepared using the LigPrep tool, assigning charges with Epik at pH 7.00 ± 1.00. The channel 3D model structures were converted to pdbqt format [51].

### 4.12. Docking and Classical Molecular Dynamics Simulations

The potential compound binding poses on Ca_V_1.2 and K_Ca_1.1 channels were obtained through a molecular docking approach, and flexible sampling was applied with the glide standard precision (SP) protocol [52]. The docking simulation was performed on 3D structures of targets obtained thorough a homology modeling procedure, due to the unavailability of 3D structures of our targets. Input charges of compounds were retained, and amide bond conformations were allowed to vary. Strain correction terms were applied to the glide scoring function and Epik state penalties were computed for the final docking score. All the other options were set to default. The Receptor Grid Generation tool from Schrödinger 2019-2 was used to generate a box able to enclose all Ca_V_1.2 and K_Ca_1.1 channels binding pocket residues. A box of 22 Å for each dimension, was generated to enclose a known blocker binding region of the Ca_V_1.2 channel [29]. While a box of 18 Å for each dimension was built for the K_Ca_1.1 channel, to involve a channel stimulator binding region. To explore the molecule interactions within the binding site of targets, the compounds were prepared by creating a phase database, minimizing the output to 100 conformers per ligand. The structures were charged according to the Epik tool at pH 7.00 ± 1.00. The specified chirality and the eight lowest-energy stereoisomers (if present) were retained. Up to 4 low-energy, 5-, and 6-membered ring conformations were generated. All high-energy conformers/tautomers were discarded. Interaction network analyses were performed using the P.L.I.P. tool [53]. A classical molecular dynamics (cMD) simulation of 100 ns was performed on the Ca_V_1.2 channel in the free state and in complex with the compounds, to investigate the potential binding mode of the compounds, as previously described [47]. In brief, CHARMM-GUI platform [17] was used to insert the system inside POPCs 128 (1-palmitoyl-2-oleoyl-glycero-3-phosphocholine) bilayer, solvated with TIP3P water models and neutralized with counter-ions. The biological system was parameterized with CHARMM-36 force field [54]. To fix all bond angles and reduce steric clashes, the energy of the system was minimized with 5.000 steps of minimization, using the steepest descent algorithm, and found to converge to a minimum energy with forces less than 100 kJ/mol/nm. Simulations were run applying periodic boundary conditions, with a cutoff radius of 1.2 nm. All the cMD simulations were performed integrating each time step of 2 fs for a run of 100 ns; a Nose–Hoover thermostat maintained the temperature at 300 K (1 ns) and a Parrinello–Rahman barostat maintained the system pressure at 1 atm (more cycles of 3 ns to achieve a stable pressure trend value), with a low dumping of 1 ps−1; the LINCS algorithm constrained the bond lengths involving hydrogen atoms. The ligands were parameterized using a CHARMM General Force Field (CGenFF), implemented in the CHARMM-GUI platform [17]. The GROMACS 2019.3 package was used to carry out and analyze the cMD trajectories [54]. PyMOL v2.5 was used as a molecular graphics system to generate the Figures (PyMOL Molecular Graphics System, New York, NY, USA, Version 2.5, Schrödinger, LLC).

## Figures and Tables

**Table 1:** Effects of compounds on KCl-induced contraction in rat aorta rings and on rat tail artery myocyte Ca_V_1.2 channel current.

Cpds	Rat Aorta Rings								Rat Tail Artery Myocytes	
	KCl				Phenylephrine				I_Ba1.2_	
	25 mM		60 mM		−Endothelium		+Endothelium			
	IC_50_ (µM)	E_max_ (%)	IC_50_ (µM)	E_max_ (%)	IC_50_ (µM)	E_max_ (%)	IC_50_ (µM)	E_max_ (%)	IC_50_ (µM)	E_max_ (%)
5	N.D.	45.9 ± 8.5 (6)	29.2 ± 6.1 (5)	73.5 ± 6.8 * (5)	N.D.	29.8 ± 13.9 (5)	N.D.	+287.1 ± 48.8 * (6)	69.0 ^a^	64.8 ± 6.1 (6)
6			11.9 ± 1.3 (5)	98.8 ± 1.3 (5)					33.2 ± 6.8 (5)	88.7 ± 4.0 (5)
7	0.2 ± 0.1 (7)	94.3 ± 4.5 (3)	9.3 ± 2.0 * (6)	89.7 ± 1.9 (5)	N.D	44.2 ± 3.8 (5)	N.D.	8.6 ± 9.3 * (6)	13.2 ± 5.4 (6)	90.5 ± 5.8 (6)
8	2.1 ± 0.9 (8)	85.8 ± 4.8 (8)	6.1 ± 1.6 * (5)	87.3 ± 4.9 (5)	44.7 ^a^	59.9 ± 10.2 (7)	N.D.	15.2 ± 7.9 * (6)	16.7 ± 3.0 (7)	92.4 ± 2.2 (7)
9	0.9 ± 0.2 (5)	92.3 ± 0.6 (5)	4.5 ± 0.5 * (5)	92.4 ± 1.3 (5)	55.0 ^a^	61.0 ± 8.9 (5)	N.D.	19.4 ± 23.2 (6)	15.3 ± 5.3 (8)	97.8 ± 0.8 (5)
10			69.2 ^a^	53.1 ± 13.4 (5)					20.8 ± 6.1 (6)	86.4 ± 4.9 (6)

Potency (expressed as IC_50_ value) and efficacy (E_max_, expressed as percent maximal inhibition) are mean ± S.E.M. (in parentheses the number of independent replicates). ^a^ Estimated value. N.D.: not detectable. * *p* < 0.05 vs. 25 mM (for KCl) or -endothelium (for phenylephrine), respectively.

**Table 2:** Effect of compound **5** derivatives on the voltage dependence of Ca_V_1.2 channel activation and inactivation.

Cpds	V_50 act_ (mV)	Slope _act_	*n*	V_50 inact_ (mV)	Slope _inact_	*n*
Control	−2.64 ± 1.03	7.16 ± 0.21	5	−21.34 ± 3.59	−12.09 ± 1.36	5
8 10 µM				−30.97 ± 3.49 *	−11.51 ± 1.90	5
8 30 µM	0.03 ± 1.35	7.61 ± 0.66	5			
Control	−1.97 ± 2.36	7.08 ± 0.32	6	−23.59 ± 1.40	−7.56 ± 0.36	6
7 10 µM	1.99 ± 3.26 *	8.36 ± 0.75	6	−30.24 ± 1.29 ^#^	−7.53 ± 0.24	6
7 30 µM				−38.29 ± 2.00 ^#^	−7.70 ± 0.51	6
Control	−2.88 ± 0.91	7.31 ± 0.27	6	−22.90 ± 2.53	−9.03 ± 1.32	5
9 10 µM	−2.37 ± 0.95	6.79 ± 0.35	6	−36.46 ± 2.73 *	−10.25 ± 1.33	5

V_50 act_: 50% activation potential; slope _act_: slope factor of activation; V_50 inact_: 50% inactivation potential; slope _inact_: slope factor of inactivation. * *p* < 0.05 vs. control, Student’s t test for paired samples; ^#^ *p* < 0.05 vs. control, repeated measures ANOVA and Dunnett post-hoc test.

**Table 3:** Effects of **7** on HR), RR, PQ, QRS, QT, and QTc in Langendorff perfused rat hearts.

(7)	HR (bpm)	RR (ms)	PQ (ms)	QRS (ms)	QTc (ms)
0	261.9 ± 9.6	231.4 ± 7.8	37.8 ± 1.8	13.4 ± 0.7	72.97 ± 1.7
1 µM	261.8 ± 9.4	230.8 ± 7.4	37.2 ± 2.0	13.6 ± 0.5	71.59 ± 0.8
3 µM	256.9 ± 8.3	231.8 ± 7.1	37.4 ± 1.9	14.0 ± 0.6	72.65 ± 1.3
10 µM	255.3 ± 8.0	236.4 ± 7.1	39.0 ± 1.9	13.9 ± 0.6	73.31 ± 1.2
30 µM	242.8 ± 11.5 **	249.8 ± 10.7 **	42.2 ± 2.7 **	15.2 ± 0.8	75.13 ± 1.7

Each value represents the mean ± s.e.m. (*n* = 5). ** *p* < 0.01, repeated measures ANOVA and Dunnet post-hoc test. HR, frequency; RR, cycle length; PQ, atrioventricular conduction time; QRS, intraventricular conduction time; QT, duration of ventricular depolarization and repolarization, i.e., the action potential duration; QTc, corrected QT.

## Data Availability

Not applicable.
